# Prostate cancer detection and complications of MRI-targeted prostate biopsy using cognitive registration, software-assisted image fusion or in-bore guidance: a systematic review and meta-analysis of comparative studies

**DOI:** 10.1038/s41391-024-00827-x

**Published:** 2024-04-05

**Authors:** Ugo Giovanni Falagario, Francesco Pellegrino, Antonio Fanelli, Francesco Guzzi, Riccardo Bartoletti, Hannes Cash, Christian Pavlovich, Mark Emberton, Giuseppe Carrieri, Gianluca Giannarini

**Affiliations:** 1 Department of Molecular Medicine and Surgery, (Solna), Karolinska Institutet, Stockholm, Sweden; 2 Department of Urology and kidney transplantation, University of Foggia, Foggia, Italy; 3 Unit of Urology/Division of Oncology, Soldera Prostate Cancer Lab, Urological Research Institute, IRCCS San Raffaele Hospital, Milan, Italy; Vita-Salute San Raffaele University, Milan, Italy; 4 Department of Translational Research and New Technologies in Medicine and Surgery, University of Pisa, Pisa, Italy; 5 Department of Urology, Otto-von-Guericke-University Magdeburg, Magdeburg, Germany; 6 PROURO, Berlin, Germany; 7 James Buchanan Brady Urological Institute and Department of Urology, Johns Hopkins University School of Medicine, Baltimore, MD USA; 8 Division of Surgery and Interventional Sciences, University College London, London, UK; 9 Department of Urology, University College London Hospital, London, UK; 10 Urology Unit, Santa Maria Della Misericordia University Hospital, Udine, Italy

**Keywords:** Prostate cancer, Diagnostic markers

## Abstract

**Background:**

Three primary strategies for MRI-targeted biopsies (TB) are available: Cognitive TB (COG-TB), MRI-US Fusion TB (FUS-TB), and In Bore TB (IB-TB). Despite nearly a decade of practice, a consensus on the preferred approach is lacking, with previous studies showing comparable PCa detection rates among the three methods.

**Methods:**

We conducted a search of PubMed, EMBASE, PubMed, Web of Science, and Scopus databases from 2014 to 2023, to identify studies comparing at least two of the three methods and reporting clinically significant PCa (csPCa) detection rates. The primary and secondary outcomes were to compare the csPCa and insignificant prostate cancer (iPCa, ISUP GG 1) detection rates between TB techniques. The tertiary outcome was to compare the complication rate between TB techniques. Detection rates were pooled using random-effect models. Planned sensitivity analyses included subgroup analysis according to the definition of csPCa and positive MRI, previous biopsy status, biopsy route, prostate volume, and lesion characteristics.

**Results:**

A total of twenty studies, involving 4928 patients, were included in the quantitative synthesis. The meta-analysis unveiled comparable csPCa detection rates among COG-TB (0.37), FUS-TB (0.39), and IB-TB (0.47). iPCa detection rate was also similar between TB techniques (COG-TB: 0.12, FUS-TB: 0.17, IB-TB: 0.18). All preplanned sensitivity analyses were conducted and did not show any statistically significant difference in the detection of csPCa between TB methods. Complication rates, however, were infrequently reported, and when available, no statistically significant differences were observed among the techniques.

**Conclusions:**

This unique study, exclusively focusing on comparative research, indicates no significant differences in csPCa and iPCa detection rates between COG-TB, FUS-TB, and IB-TB. Decisions between these techniques may extend beyond diagnostic accuracy, considering factors such as resource availability and operator preferences. Well-designed prospective studies are warranted to refine our understanding of the optimal approach for TB in diverse clinical scenarios.

## Introduction

The advent of magnetic resonance imaging (MRI) and target biopsies (TB) directed at MRI-suspicious lesions has revolutionized the accuracy of Prostate cancer (PCa) diagnosis and biopsy sampling. Indeed, level 1 evidence suggests that this pathway enhances the diagnosis of clinically significant PCa (csPCa) while mitigating the risk of overdiagnosing indolent PCa (iPCa) [1–3]. Notably, the incorporation of target sampling alongside standard prostate biopsies achieves higher concordance between biopsy results and final pathology examination on whole gland specimens [4–6].

Three distinct strategies for conducting MRI-TB have emerged, each accompanied by its own set of advantages and potential limitations. Cognitive target biopsies (COG-TB) rely on the urologist’s capacity to mentally co-register MRI findings with ultrasound (US) images, demanding a high level of expertise and familiarity with both imaging modalities. In contrast, MRI-US Fusion target biopsies (FUS-TB) employ software capable of overlaying 3D reconstructions from MRI onto real-time US images, guiding the biopsy needle to suspicious areas within the prostate. Lastly, In Bore targeted biopsies (IB-TB) utilize an MRI-compatible device with the patient positioned inside the MRI scanner, enabling direct visualization of the suspicious lesion, needle guide, and biopsy needle throughout the sampling process.

Although these strategies have been practiced for almost a decade, a consensus on the preferred approach to TB is currently lacking [7]. Three randomized controlled trials (RCT) showed similar PCa detection rates between IB-TB, FUS-TB, and COG-TB [8–10]. Similarly, the most recent systematic reviews and metanalysis showed no difference between the three techniques [11, 12]. However, the metanalysis was not restricted to comparative studies, and significant differences in baseline characteristics in each cohort might have potentially limited the validity of the findings.

With this in mind, we conducted a systematic review and meta-analysis including only comparative studies between IB-TB, FUS-TB, and COG-TB aiming to evaluate which of the MRI-TB method has the highest diagnostic yield for csPCa and the lowest risk of overdiagnosis of iPCa and complications.

## Materials/subjects and methods

### Search strategy

A comprehensive systematic review of the literature was conducted by searching the EMBASE, PubMed, Web of Science, Scopus and Cochrane Library (CENTRAL) databases.

Research terms used for the research were the following: “(prostate cancer OR prostate adenocarcinoma) AND (MRI OR magnetic resonance) AND (target* OR biopsy)”.

We searched from January 2014 up to November 1, 2023. All the references of included manuscripts and previous reviews were also screened [11, 12]. This systematic review was reported in compliance to the Preferred Reporting Items for Systematic Reviews and Meta-analyses protocol (PRISMA) [13] and was registered within the international prospective registry of systematic reviews (PROSPERO, CRD42024501439).

### Initial screening, eligibility/Inclusion criteria

After identifying the initial set of studies, a reviewer (FP) undertook the removal of duplicate entries. Subsequently, two reviewers (FG, AF) assessed independently all the titles and abstracts (and full text, in need of further clarification) for relevance. The eligibility of studies and data extraction were performed with a comprehensive full-text review conducted by two reviewers (FG, AF). In instances of disagreement, a consensus was reached through consultation with a third reviewer (UF).

The population, intervention, comparator, and outcome (PICO) approach [14] was used to define the research question and study eligibility as follows: In patients with a positive MRI (P), what is the best target biopsy (I) technique between cognitive registration, software-assisted image fusion or in-bore sampling fusion (C) to detect clinically significant prostate cancer?(O).

Studies that met the following criteria were eligible for quantitative synthesis and meta-analysis: (A) comparative studies between at least two of the three MRI-TB methods, (B) available detection rate for csPCa by MRI-TB method, (C) mpMRI performed and reported according to ESUR or PIRADS v1 or v2 criteria, (D) available definition of csPCa. Duplicated studies, studies not providing data of the outcomes of interest, review articles, case reports, letters, or conference abstracts were excluded.

### Definition of outcomes, data extraction, and quality assessment

The primary outcome of the study was the pooled detection rate of csPCa at MRI-TB using different MRI-TB techniques. Since there is no universally accepted definition of csPCa, definitions used in individual studies were used. The detection rate was defined as the proportion of patients who underwent TB with csPCa at TB. The secondary outcome was the pooled detection rate of iPCa defined as ISUP Grade Group (ISUP GG = 1). Finally, the tertiary outcome was high-grade complications (Clavien-Dindo ≥2) [15].

The number of patients diagnosed with csPCa and iPCa in each study arm was extracted together with the definitions of the outcomes used in each study. When reported, we also extracted the number of patients diagnosed with each ISUP Grade Group.

Study characteristics, study time frame, details for MRI acquisition and reporting, and baseline data of included patients were extracted from each included study. Baseline data included Age, PSA, Prostate volume, PIRADS scores, number of patients with a previous negative prostate biopsy, and number of patients on active surveillance. When available, baseline data were extracted for the overall population and according to each of the study cohorts. The methodological quality of the studies was evaluated using the Quality Assessment of Diagnostic Accuracy Studies-2 (QUADAS-C) tool [16]. The assessment was performed by 2 reviewers (AF, FG) and checked by a second (UF). QUADAS-C is a tool recommended for use in systematic reviews to evaluate the risk of bias and the applicability of comparative diagnostic accuracy studies [16].

### Statistical analysis

First, a summary table with study characteristics was created.

Then, we employed the accuracy measurements as previously defined and specifically targeted studies that reported one of the MRI-TB techniques, namely IB-TB, FUS-TB, or COG-TB. We synthesized pooled estimates by performing random-effect meta-analyses. All results were reported with 95% confidence intervals (CI). The I^2^ statistic [17] and the between-study variance (t^2^) from the random-effect analysis were used to quantify the heterogeneity between the studies. I^2^ values > 50% indicated large heterogeneity. All models have allowed for different detection rates (random effects) unless otherwise specified. In case of large heterogeneity, random-effect models (using the DerSimonian and Laird approach [18]) were prioritized. A meta-analysis of single means between studies for continuous variables was performed using the inverse variance method for pooling.

Preplanned sensitivity analyses included subgroup analysis according to study design (including only RCTs) definition of csPCa (including studies reporting ISUP GG ≥ 2 detection rates), definition of positive MRI (studies including patients with PI-RADS/Likert score ≥3), previous biopsy status (biopsy naïve vs previous negative prostate biopsy), biopsy route (transrectal vs transperineal), prostate volume (≤50 ml vs >50 ml), target lesion location (Peripheral zone vs Transition Zone) and target lesion size (≤10 mm vs >10 mm). Finally, we repeated our analyses using as outcome the detection of csPCa at biopsies overall. For this analysis, if a patient underwent MRI-TB plus SB the outcome was the detection of csPCa at combined biopsies, whereas if the patient underwent only MRI-TB the outcome was the detection of csPCa at MRI-TB. The extracted data were computed and pre-calculated in Microsoft Excel, while the meta-analyses were executed in R Studio Version 1.2.1335 (Boston, MA, USA).

## Results

### Study characteristics

**Fig. 1:**
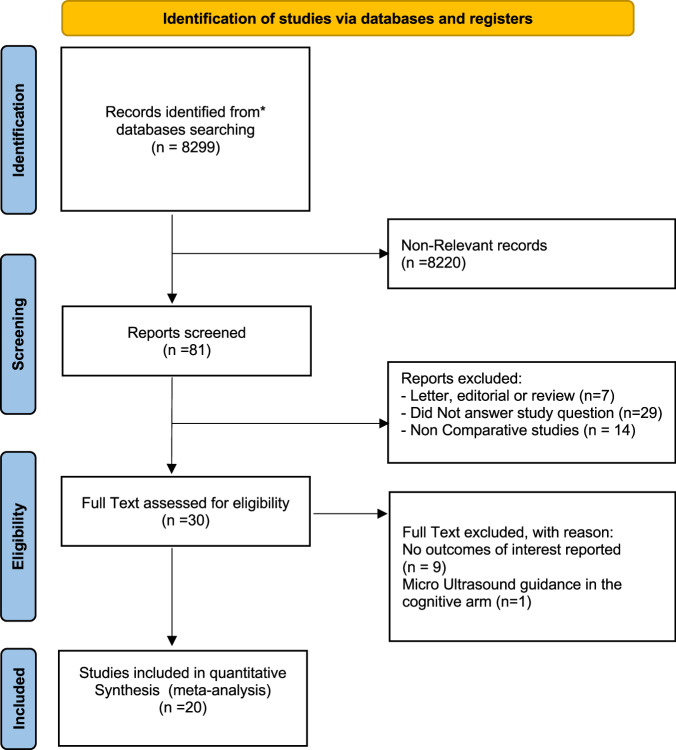
Preferred reporting items for systematic reviews and meta-analysis (PRISMA) flow chart.

Twenty studies were deemed eligible for the quantitative analyses including a total of 4928 patients (1931 COG-TB, 2432 FUS-TB and 1050 IB-TB). PRISMA flow chart is presented in Fig. 1.

Study design, details for MRI acquisition and reporting, and baseline data of included patients are presented in Table 1. Eight of the included studies were prospective including three RCTs. Four studies had a within patient’s comparison design with each patient undergoing each of the MRI-TB technique [9, 19, 20]. In one of these the order for preforming TB was randomized [9]. Three studies compared all three MRI-TB modalities [10, 21, 22] while most studies (*n* = 17) compared any combination of two techniques. The mean age ranged from 59 to 72 years and the mean PSA value ranged from 4.2 ng/ml to 14.1 ng/ml. The populations varied with respect to the inclusion of patients with previous biopsy, 6 and 5 studies respectively included only biopsy naïve and previous negative biopsy patients. The remaining (*n* = 9) included mixed cohorts with three also including patients with a previous positive biopsy with iPCa on active surveillance [9, 19, 23]. PIRADS score was the preferred reporting system in 18 studies (PIRADSv2 in 12) and 3 Tesla (T) MRI scanners were used in 14 studies. Results of MRI-TB only were reported by most studies except 4 that only reported the final Gleason score from the combination of Target and Random cores.

**Table 1:** Baseline characteristics and applied methodology of included studies.

Author, yr of Publication	Study design	Biopsy setting	MRI magnet strength, scoring system used	Threshold for biopsy	csPCa definition	Biopsy Tech	No. of patients	csPCa DR TB only (*n*, %)	csPCa DR TB+ random (*n*, %)	Mean age (yr)	Mean PSA (ng/ml)
Turkay et al. [31]	Retrospective, unpaired	Biopsy naive	N/A, PI-RADSv N/A	≥4	≥3 + 4	COG-TB	50	N/A	15, 30%	59.2	5.4
						FUS-TB	50	N/A	18, 36%	58	5.5
Zhang et al. [34]	Retrospective, unpaired	Biopsy naive	1,5 T(COG) 3 T(IB), PI-RADSv2	≥2	≥3 + 4 or 5 mm 3 + 3	COG-TB	85	20, 23%	24, 28%	63	7.4
						IB-TB	88	26, 29%	N/A	70	6.8
Yamada et al. [33]	Retrospective, unpaired	Biopsy naive	3 T, PI-RADSv2	≥3	≥3 + 4	COG-TB	113	N/A	82, 73%	72	8.3
						FUS-TB	185	N/A	140, 76%	70	7.9
Izadpanahi et al. [30]	Prospective, randomized	Biopsy naive	3 T, PI-RADSv2	≥4	≥3 + 4 or 4 mm 3 + 3	COG-TB	100	17, 17%	19, 19%	61.9	5.9
						FUS-TB	99	30, 30%	33, 33%	61.9	6.1
Khoo et al. [32]	Retrospective, unpaired	Biopsy naive	3 T, PI-RADSv2	≥4	≥3 + 4	COG-TB	372	201, 54%	N/A	67.3	7.7
						FUS-TB	699	401, 57%	N/A	67.2	7.4
Ito et al. [36]	Retrospective, unpaired	Biopsy naive	N/A, PI-RADSv2	≥4	≥3 + 4 or 5 mm 3 + 3	COG-TB	97	46, 47%	68, 70%	71	7.2
						FUS-TB	74	57, 77%	70, 96%	70	7.5
Arsov et al. [8]	Prospective, randomized	Repeat biopsy	3 T, PI-RADSv1	>3	≥3 + 4	FUS-TB	104	26, 25%	32, 31%	68	10.8
						IB-TB	106	29, 27%	N/A	66	10
Venderink et al. [26]	Retrospective, unpaired	Repeat biopsy	3 T, PI-RADSv1-v2	≥4	≥3 + 4	FUS-TB	51	25, 49%	N/A	69	11
						IB-TB	227	139, 61%	N/A	67	12.8
Simmons LAM et al. [24]	Prospective, within-person paired	Repeat biopsy	3 T, LIKERT	≥3	≥4 + 3 or 6 mm 3 + 4	COG-TB	169	104, 61%	70, 41%	62.6	7.6
						FUS-TB	169	108, 64%	74, 44%		
Kaufmann et al. [21]	Prospective, unpaired	Repeat biopsy	3 T, PI-RADSv2	≥4	≥3 + 4 or 5 mm 3 + 3	COG-TB	38	9, 24%	N/A	69	9
						FUS-TB	73	30, 41%	N/A	67	9
						IB-TB	45	18, 40%	N/A	64	9
Hamid et al. [9]	Prospective, within-person randomized paired	Repeat biopsy	1.5 T, LIKERT	≥3	≥3 + 4 or 4 mm 3 + 3	COG-TB	129	68, 53%	N/A	65	8.5
						FUS-TB	129	69, 53%	N/A	65	8.5
Wegelin et al. [10]	Prospective, randomized	Repeat biopsy	3 T, PI-RADSv2	≥3	≥3 + 4	COG-TB	78	26, 33%	N/A		
						FUS-TB	79	27, 34%	N/A	65.7	6.4
						IB-TB	77	25, 33%	N/A		
Wysock JS et al. [19]	Prospective, within-person paired	Mixed	3 T, LIKERT	≥2	≥3 + 4	COG-TB	125	24, 19%	N/A	65	5.1
						FUS-TB	125	29, 23%	N/A	65	5.1
Oderda et al. [35]	Retrospective, unpaired	Mixed	1.5 T, PI-RADSv1	≥3	N/A	COG-TB	25	7, 28%	15, 60%	62	8.4
						FUS-TB	25	3, 12%	7, 28%	64	7.9
Oberlin et al. [28]	Retrospective, unpaired	Mixed	3 T, PI-RADSv1	≥3	≥3 + 4	COG-TB	150	N/A	25, 17%	64.9	4.26
						FUS-TB	81	N/A	24, 30%	64.3	5.1
Yaxley et al. [27]	Retrospective, unpaired	Mixed	3 T, PI-RADSv1	≥4	5 mm 3 + 4 or 6 mm 3 + 3	COG-TB	194	163, 84%	N/A	64.9	5.6
						IB-TB	249	203, 81%	N/A	64.4	5.6
Osses et al. [25]	Retrospective, unpaired	Mixed	3 T, PI-RADSv1	≥2	≥3 + 4	COG-TB	64	20, 31%	32, 50%	68	14.3
						IB-TB	155	63, 41%	N/A	68	11.1
Costa et al. [23]	Retrospective, unpaired	Mixed	3 T, PI-RADSv2	≥3	≥3 + 4	FUS-TB	300	141, 47%	N/A	68	8.6
						IB-TB	103	63, 61%	N/A	64	8
Guerra-Lacambra et al. [29]	Retrospective, unpaired	Mixed	1.5 T, PI-RADSv2	≥4	≥3 + 4	COG-TB	40	N/A	4, 10%	66	5.3
						FUS-TB	87	N/A	17, 19%	67	5.2
Petov et al. [20]	Prospective, within-person paired	Mixed	3 T, PI-RADSv2	≥3	≥3 + 4	COG-TB	102	31, 30%	N/A	63.5	6.7
						FUS-TB	102	29, 28%	N/A	65.5	6.7

### Quality assessment

Quality was evaluated for studies included in the meta-analysis (*n* = 20) (Supplementary Table 1). All studies were estimated to have low risk regarding applicability to the current review. Thirteen studies were deemed to have a high risk of selection bias.

#### Diagnosis of csPCa, and iPCa at MRI-TB according to MRI-TB technique

**Fig. 2:**
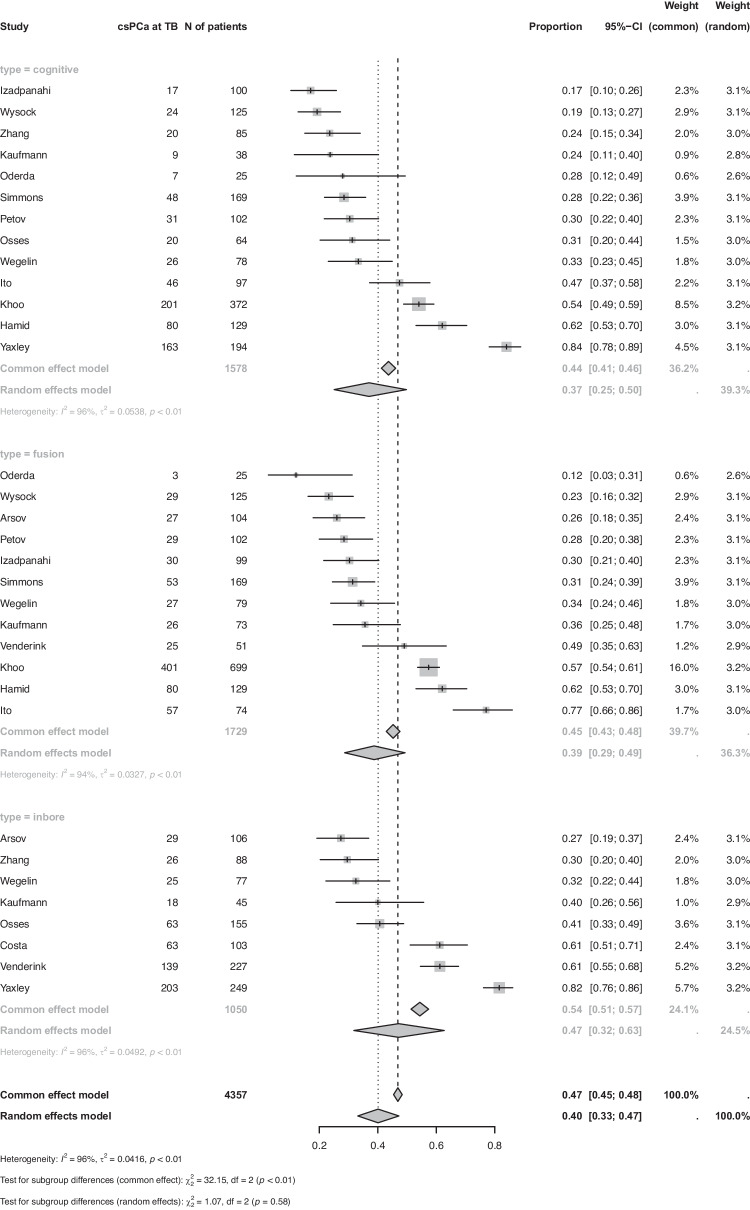
Detection rate of clinically significant prostate cancer at targeted biopsy according to targeting technique. CsPCa at TB: clinically significant prostate cancer at targeted biopsy; N of patients: number of patients.

For the primary outcome (csPCa at MRI-TB), data were extracted from 13 studies for COG-TB (1578 patients), 12 studies for FUS-TB (1729 patients), and 8 studies for IB-TB (1050 patients). Pooled meta-analyses with random-effect models demonstrated a csPCa detection rate of 0.37 (CI: 0.25; 0.50) for COG-TB, 0.39 (CI: 0.29; 0.49) for FUS-TB, and 0.47 (CI: 0.32, 0.63) for IB-TB (Fig. 2).

**Fig. 3:**
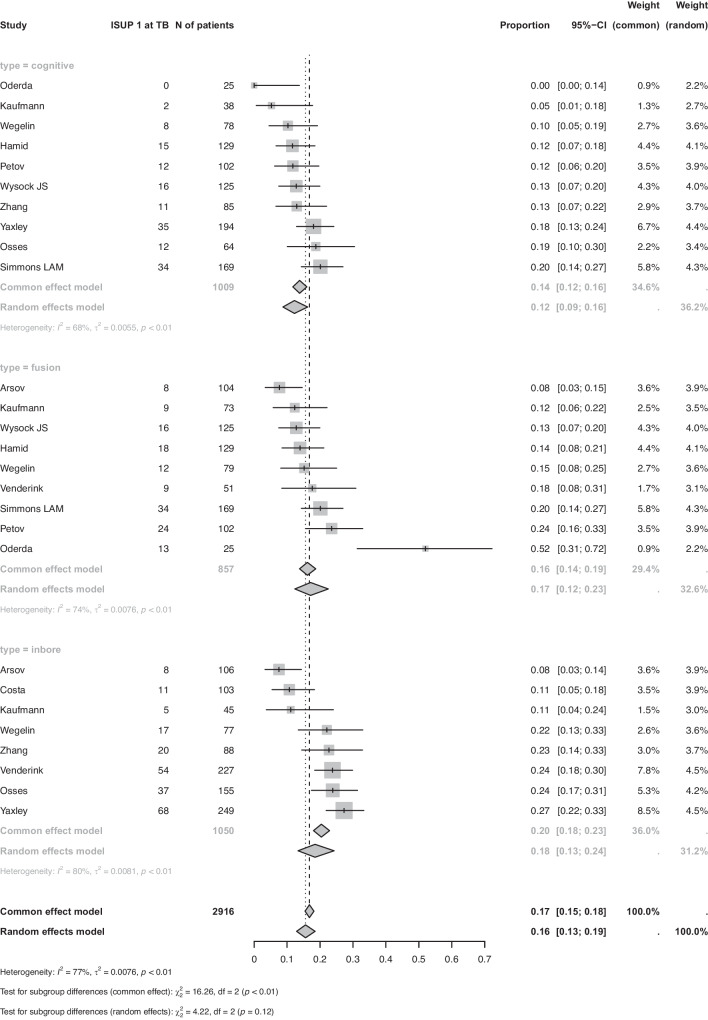
Detection rate of indolent prostate cancer (ISUP Grade Group 1) at targeted biopsy according to targeting technique. N of patients: number of patients; ISUP 1 at TB: indolent prostate cancer at targeted biopsy.

Ten studies for COG-TB (1009 patients), 9 studies for FUS-TB (857 patients), and 8 studies for IB-TB (1050 patients) reported data to evaluate the secondary outcome (iPCa at MRI-TB). Pooled meta-analyses with random-effect models demonstrated iPCa detection rate of 0.12 (CI: 0.09; 0.16) for COG-TB, 0.17 (CI: 0.12; 0.23) for FUS-TB, and 0.18 (CI: 0.13; 0.24) for IB-TB (Fig. 3). All analyses were characterized by significant heterogeneity (I^2^ > 77%, *p* < 0.01). The differences between MRI-TB techniques were not statistically different at univariable metaregression (*p* > 0.05). Since Funnel plots showed evidence for a potential publication bias (Supplementary Fig. 2), we performed a sensitivity analysis including only studies with >100 patients and >50 anyPCa cases showing a statistically significant difference in only the detection rate of anyPCa (*p*: 0.05) (Supplementary Fig. 3). Indeed, this was 0.79 (CI: 0.52; 0.97) for COG-TB, 0.62 (CI: 0.53; 0.70) for FUS-TB, and 0.79 (CI: 0.66; 0.89) for IB-TB.

The sensitivity analysis including only RCTs did not show any differences in the diagnosis of csPCa at MRI-TB between MRI-TB techniques (Supplementary Fig. 4).

### Complications

Out of the 20 studies included, 4 had a within patients design and were excluded for the evaluation of complications according to MRI-TB method [9, 19, 20, 24]. Eleven studies did not comment on complications [23, 25–34]. The remaining 5 studies [8, 10, 21, 35, 36], including a total of 821 patients, reported 19 (2.3%) Grade 2 adverse events. Out of these, 11 were infective complications (with 7 requiring hospitalization), 4 urinary tract symptoms progression for which treatment was initiated. Two studies reported no significant complications [21, 35]. None of the studies reported any difference between groups in terms of complication except for the FUTURE trial in the setting of repeat biopsy. In the latter, a lower rate of minor adverse events was noted in patients who underwent IB-TB that was likely caused by the omission of standard biopsies [37].

#### Preplanned sub-analysis

##### Definition of csPCa (ISUP GG ≥ 2)

Seven studies did not define csPCa as ISUP GG ≥ 2 [9, 21, 24, 27, 30, 34, 36]. Among these, 4 studies provided data to evaluate ISUP GG ≥ 2 at MRI-TB as outcome [9, 21, 24, 27]. Therefore, data were extracted from 10 studies for COG-TB (1009 patients), 9 studies for FUS-TB (857 patients), and 8 studies for IB-TB (1050 patients). Pooled meta-analyses with random-effect models demonstrated csPCa detection rate of 0.36 (CI: 0.23; 0.50) for COG-TB, 0.35 (CI: 0.27; 0.43) for FUS-TB, and 0.45 (CI: 0.34; 0.55) for IB-TB (Supplementary Fig. 5), with large heterogeneity (I^2^ > 93%, *p* < 0.01). This difference was not statistically significant at univariable metaregression (*p* > 0.05).

##### Definition of positive MRI (PI-RADS/Likert score ≥ 3)

Only 8 studies defined positive MRI as PI-RADS ≥ 3 [9, 10, 20, 23, 24, 28, 31, 33]. Therefore, we considered only these studies for the next steps. For these analyses, data were extracted from 4 studies for COG-TB (478 patients), 4 studies for FUS-TB (479 patients), and 2 studies for IB-TB (180 patients). The pooled detection rate of csPCa were 0.38 (CI: 0.23; 0.55) for COG-TB, 0.39 (CI: 0.24; 0.55) for FUS-TB, and 0.47 (CI: 0.20; 0.74) for IB-TB at meta-analyses with random-effect models (Supplementary Fig. 6), with significant heterogeneity (I^2^ 51%, p: 0.03) and no statistically significant difference at univariable metaregression (*p* > 0.05).

##### Previous biopsy status

We repeated our analysis in studies including only biopsy-naive patients and previous negative biopsy patients or studies reporting their results separately for patients with different biopsy histories. Five studies for COG-TB (721 patients), 4 studies for FUS-TB (939 patients), and 1 study for IB-TB (88 patients) reported results on biopsy naïve patients and had complete data to evaluate the detection of csPCa. Pooled meta-analyses with random-effect models demonstrated a csPCa detection rate of 0.33 (CI: 0.18; 0.50) for COG-TB, 0.48 (CI: 0.29; 0.68) for FUS-TB, and 0.30 (CI: 0.20, 0.40) for IB-TB (Supplementary Fig. 7A), with large heterogeneity (I^2^ 95%, *p* < 0.01).

Four study for COG-TB (319 patients), 6 studies for FUS-TB (510 patients), and 4 studies for IB-TB (455 patients) reported results on previous negative biopsy patients and had complete data to evaluate the detection of csPCa. Pooled meta-analyses with random-effect models demonstrated a csPCa detection rate of 0.26 (CI: 0.20; 0.34) for COG-TB, 0.33 (CI: 0.26; 0.39) for FUS-TB, and 0.40 (CI: 0.23, 0.59) for IB-TB (Supplementary Fig. 7B), with significant heterogeneity (I^2^ 86%, *p* < 0.01). In both cases, we did not find a statistically significant difference in csPCa detection rate between MRI-TB techniques at univariable metaregression (*p* > 0.05).

##### Biopsy route (transrectal vs transperineal)

Five studies for COG-TB (392 patients), 6 studies for FUS-TB (483 patients), and 5 study for IB-TB (668 patients) reported results on transrectal MRI-TB and had complete data to evaluate the detection of csPCa. Pooled meta-analyses with random-effect models demonstrated a csPCa detection rate of 0.25 (CI: 0.18; 0.32) for COG-TB, 0.29 (CI: 0.22; 0.37) for FUS-TB, and 0.45 (CI: 0.31, 0.59) for IB-TB (Supplementary Fig. 8A), with large heterogeneity (I^2^ 91%, *p* < 0.01). This difference was statistically significant (*p*: 0.04).

None of the studies reported results of transperineal IB-TB and we included in the subanalysis 5 studies for transperineal COG-TB (869 patients) and 5 studies for transperineal FUS-TB (1173 patients) (Supplementary Fig. 8B). csPCa detection rates were 0.44 (CI: 0.32; 0.57) and 0.51 (CI: 0.36; 0.66) respectively (I^2^ 94%, *p* < 0.01). Univariable metaregression did not show any statistically significant difference in csPCa detection rate according to biopsy route (*p* > 0.05).

##### Prostate volume, target lesion location, and target lesion size

Four studies reported subgroups csPCa detection rates according to prostate volume, target lesion location, and target lesion size [10, 23, 32, 33]. Since there was no univocal cutoff for prostate volume subanalysis, preplanned meta-analyses were performed only according to target lesion location (Peripheral zone lesions vs transitional zone lesions Supplementary Fig. 9A, B) and target lesion size (≤10 mm vs >10 mm, Supplementary Fig. 10A, B). No difference was found in csPCa detection rates at univariable metaregression (*p* > 0.05).

##### Combined biopsy

Finally, we tested the difference in the detection of csPCa at a combined approach (MRI-TB plus SB). Data for this analysis were available from 9 studies for COG-TB (724 patients), 9 studies for FUS-TB (1005 patients), and 8 studies for IB-TB (1050 patients). All patients underwent MRI-TB and SB, but those undergoing IB-TB received only MRI-TB. Pooled meta-analyses with random-effect models demonstrated a csPCa detection rate of 0.38 (CI: 0.22; 0.56) for COG-TB, 0.45 (CI: 0.28; 0.62) for FUS-TB, and 0.47 (CI: 0.32, 0.63) for IB-TB (Supplementary Fig. 11), with significant heterogeneity (I^2^ 96%, *p* < 0.01). This difference was not significant at univariable metaregression (*p* > 0.05)

## Discussion

In this systematic review and meta-analysis comparing COG-TB, FUS-TB, and IB-TB we found no significant differences in the detection rates of csPCa among the three biopsy techniques. Study results were consistent in all preplanned subgroup analysis.

Current knowledge comparing MRI-TB techniques is limited. The FUTURE trial is the only three-arm RCT so far available in men undergoing repeat biopsy and showed no difference in PCa and csPCa detection among the three techniques [10].

The presence of several confounding factors makes any comparison extremely challenging and may limit the validity of the latest meta-analysis including non-comparative reports [11, 12].

Our study aimed to contribute insights into the ongoing debate on the optimal approach for MRI-TB by including only comparative studies and thus reducing the potential risk of inclusion bias.

COG-TB results are intuitively affected by a longer learning curve. Similarly, in a patient with a small MRI lesion within a big prostate, the probabilities of hitting the target via COG-TB or FUS-TB are lower [38]. In our preplanned subgroup analysis, we did not find any difference in csPCa detection rates based on lesion characteristics. However, we could only compare 4 studies reporting subgroup’s CDRs and we only stratified the analysis according to lesion size (<10 mm) and lesion location (PZ vs TZ). It may be relevant to stratify for other lesion-specific factors such as the presence of anterior vs posterior lesions. Indeed, Wysock et al. demonstrated some benefits associated with a FUS-TB for anterior tumors, but that study was conducted using a transrectal approach, which might have made the sampling of these tumors that were furthest away from the needle deployment subject to some systematic error [19]. Only one study compared csPCa CDR in anterior or posterior lesions showing no statistically significant differences. However, these subanalyses should be interpreted with caution due to the small sample size [10].

The choice between these strategies may be influenced by factors other than diagnostic accuracy, such as resource availability, cost-effectiveness, and operator preferences.

An important consideration may be the difference in complication rates between the three MRI-TB. In the present study, we attempted to evaluate if any difference in complication rates could justify the choice of one MRI-TB technique over the others. None of the studies was specifically designed with complications as primary outcome and 5 out of 20 studies [8, 10, 21, 35, 36] reported adverse events according to biopsy techniques. A post-hoc analysis of the FUTURE trial showed a higher low-grade complication rate in patients undergoing FUS-TB or COG-TB compared to IB-TB [37]. A possible explanation is the lowest number of cores needed with the IB biopsy technique. Indeed, standard sampling is not usually performed in patients undergoing IB-TB while it is considered standard of care in patients undergoing COG-TB and FUS-TB. Additionally, IB-TB in all the included studies was performed by a transrectal approach that is inherently associated with a higher risk of infective complications [39].

It is crucial to acknowledge the limitations of our study, which may restrict the overall generalizability of our findings.

First, the high heterogeneity among the included studies reflects the absence of strict guidelines on performing prostate biopsies in patients with suspicious MRI lesions.

Second, there is a potential for publication bias, as most studies are published by high-volume centers with significant expertise.

Third, despite our efforts to reduce inclusion bias by excluding non-comparative studies, the evidence quality remains low. Most studies fail to adjust for all possible confounders that can impact the clinically significant cancer detection rate at the target biopsy. Additionally, we were unable to perform some preplanned sub-analyses stratifying by PIRADS score and the number of target cores.

It is noteworthy that the included studies not only compare MRI-targeted biopsy techniques but also assess the accuracy of MRI and the indications for prostate biopsy. These three aspects are interconnected with the accuracy of the diagnosis and should be standardized in future studies addressing this issue. MRI and MRI-TB indications should be explicitly stated in the study protocol, enrolling consecutive series of patients selected for MRI [11]. MRI acquisition and reporting should adhere to the latest guidelines and be performed by the same radiologists [40]. A standardized biopsy protocol should be adopted, ensuring an equal number of cores taken at MRI-TB, consistent biopsy routes, and uniform pathology evaluation.

Some of the variability in CDRs between techniques can be attributed to the skill of the urologist or radiologist performing the biopsy. These may impact each of the TB techniques differently and future studies should aim to compare the learning curves for each biopsy method [41].

Finally, target biopsy platforms and imaging may completely change over the next years. Indeed, artificial intelligence is showing promising results for image analysis, real time delineation of suspicious lesions on ultrasound images, and elastic registration between ultrasound and MRI [42].

Similarly, novel imaging techniques such as PSMA PET/CT have been proposed to further improve patient selection for prostate biopsy [43] and new methods to target PSMA PET/CT findings are under evaluation [44].

## Conclusion

Our study adds to the ongoing debate on the optimal approach for MRI-TB by providing a comprehensive comparison of COG-TB, FUS-TB, and IB-TB. With the caveat of heterogeneity and suboptimal quality of the included studies, the lack of significant differences in csPCa detection rates among the techniques may suggest that the choice between them may be influenced by factors beyond diagnostic accuracy, such as resource availability, cost-effectiveness, and operator preferences. Further research, including well-designed prospective studies, is warranted to address the limitations of current evidence and refine our understanding of the optimal approach for MRI target biopsies in diverse clinical scenarios.

## Supplementary information

Supplemental Material
