# Supplementary material for: Prostate cancer detection and complications of MRI-targeted prostate biopsy using cognitive registration, software-assisted image fusion or in-bore guidance: a systematic review and meta-analysis of comparative studies
Source: Prostate Cancer Prostatic Dis. 2024 Apr 5;28(2):270–9. doi: 10.1038/s41391-024-00827-x (PMC12106061; doi:10.1038/s41391-024-00827-x)
Supplement: Supplementary file 1 — Supplemental Material [file 41391_2024_827_MOESM1_ESM.docx]

**Prostate cancer detection and complications of MRI-targeted prostate biopsy using cognitive registration, software-assisted image fusion or in-bore guidance: a systematic review and meta-analysis of comparative studies**

**Supplementary material**

**sTable 1:** QUADAS-2 judgments to the left and QUADAS-C judgments to the right.

**sFigure 1.** Funnel plot to visually detect the presence of publication bias or heterogeneity for studies reporting cancer detection rates of clinically significant PCa

**sFigure 2.** Funnel plot to visually detect the presence of publication bias or heterogeneity for studies reporting cancer detection rates of insignificant PCa

**sFigure 3A.** Detection rate of clinically significant prostate cancer (csPCa)(3A) and indolent prostate cancer (ISUP GG 1) (3B) at targeted biopsy according to targeting technique.

**sFigure 4.** Detection rate of clinically significant prostate cancer (csPCa) at targeted biopsy according to targeting technique in randomized controlled studies. CsPCa at TB: clinically significant prostate cancer at targeted biopsy; N of patients

**sFigure 5.** Detection rate of ISUP Grade Group (ISUP GG) ≥2 at targeted biopsy according to target technique. CsPCa at TB: clinically significant prostate cancer at targeted biopsy; N of patients: number of patients; ISUP 1 at TB: indolent prostate cancer at targeted biopsy.

**sFigure 6.** Detection rate of clinically significant prostate cancer (csPCa)(6A) and indolent prostate cancer (ISUP GG 1) (6B) at targeted biopsy according to targeting technique in studies including all patients with PIRADS ≥3.

CsPCa at TB: clinically significant prostate cancer at targeted biopsy; N of patients: number of patients; ISUP 1 at TB: indolent prostate cancer at targeted biopsy.

**sFigure 7**. Detection rate of clinically significant prostate cancer (csPCa) at targeted biopsy according to targeting technique in biopsy naïve patients (7A) and patients with a previous negative biopsy (7B). CsPCa at TB: clinically significant prostate cancer at targeted biopsy; N of patients: number of patients.

**sFigure 8.** Detection rate of clinically significant prostate cancer (csPCa) at targeted biopsy according to targeting technique in patients undergoing transrectal targeted biopsy (8A) or transperineal targeted biopsy (8B). CsPCa at TB: clinically significant prostate cancer at targeted biopsy; N of patients: number of patients.

**sFigure 9.** Detection rate of clinically significant prostate cancer (csPCa) at targeted biopsy according to targeting technique in patients in patients with Peripheral zone (9A) or Transition Zone MRI lesions (9B). CsPCa at TB: clinically significant prostate cancer at targeted biopsy; N of patients: number of patients.

**sFigure 10.** Detection rate of clinically significant prostate cancer (csPCa) at targeted biopsy according to targeting technique in patients in patients with ≤10 mm (10A) or >10 mm (10B) MRI lesions. CsPCa at TB: clinically significant prostate cancer at targeted biopsy; N of patients: number of patients.

**sFigure 11.** Detection rate of clinically significant prostate cancer (csPCa) at a combination of targeted and Systematic Biopsy according to targeting technique. CsPCa at combined biopsy: clinically significant prostate cancer at combined standard+targeted biopsy (for patients undergoing in bore-TB only targeted cores are considered); N of patients: number of patients.

**sTable 1:** QUADAS-2 judgments to the left and QUADAS-C judgments to the right.

| **Study** | **Test** | **Risk of bias**  **(QUADAS-2)** | | | | **Applicability concerns**  **(QUADAS-2)** | | | | **Risk of bias**  **(QUADAS-C)** | | | | |
| --- | --- | --- | --- | --- | --- | --- | --- | --- | --- | --- | --- | --- | --- | --- |
|  |  | **P** | **I** | **R** | **FT** |  | **P** | **I** | **R** |  | **P** | **I** | **R** | **FT** |
| Hamid et al., 2019 | COG-TB | ✓ | ✓ | ✓ | ✓ |  | ✓ | ✓ | ✓ |  | ✓ | ✓ | ✓ | ✓ |
|  | FUS-TB | ✓ | ✓ | ✓ | ✓ |  | ✓ | ✓ | ✓ |  |  |  |  |  |
| Wysock JS et al., 2014 | COG-TB | ✓ | ✓ | ✓ | ✓ |  | ✓ | ✓ | ✓ |  | ✓ | ✓ | ✓ | ✓ |
|  | FUS-TB | ✓ | ✓ | ✓ | ✓ |  | ✓ | ✓ | ✓ |  |  |  |  |  |
| Arsov et al., 2015 | FUS-TB | ✓ | ✓ | ✓ | ✓ |  | ✓ | ✓ | ✓ |  | ✓ | ✓ | ✓ | ✓ |
|  | IB-TB | ✓ | ✓ | ✓ | ✓ |  | ✓ | ✓ | ✓ |  |  |  |  |  |
| Oderda et al., 2016 | COG-TB | ✗ | ✓ | ✓ | ✓ |  | ✓ | ✓ | ✓ |  | ✗ | ✓ | ✓ | ✓ |
|  | FUS-TB | ✗ | ✓ | ✓ | ✓ |  | ✓ | ✓ | ✓ |  |  |  |  |  |
| Oberlin et al., 2016 | COG-TB | ✗ | ✓ | ✓ | ✓ |  | ✓ | ✓ | ✓ |  | ✗ | ✓ | ✓ | ✓ |
|  | FUS-TB | ✗ | ✓ | ✓ | ✓ |  | ✓ | ✓ | ✓ |  |  |  |  |  |
| Venderink et al., 2017 | FUS-TB | ✗ | ✗ | ✓ | ? |  | ✓ | ✓ | ✓ |  | ✗ | ✓ | ✓ | ? |
|  | IB-TB | ✗ | ✗ | ✓ | ? |  | ✓ | ✓ | ✓ |  |  |  |  |  |
| Yaxley et al., 2017 | COG-TB | ✗ | ✓ | ✓ | ✓ |  | ✓ | ✓ | ✓ |  | ✗ | ✓ | ✓ | ✓ |
|  | IB-TB | ✗ | ✓ | ✓ | ✓ |  | ✓ | ✓ | ✓ |  |  |  |  |  |
| Simmons LAM et al. 2018 | COG-TB | ✓ | ✓ | ✓ | ✓ |  | ✓ | ✓ | ✓ |  | ✓ | ✓ | ✓ | ✓ |
|  | FUS-TB | ✓ | ✓ | ✓ | ✓ |  | ✓ | ✓ | ✓ |  |  |  |  |  |
| Osses et al., 2018 | COG-TB | ✗ | ✓ | ✓ | ✓ |  | ✓ | ✓ | ✓ |  | ✗ | ✓ | ✓ | ✓ |
|  | IB-TB | ✗ | ✓ | ✓ | ✓ |  | ✓ | ✓ | ✓ |  |  |  |  |  |
| Kaufmann et al., 2018 | COG-TB | ✗ | ✓ | ✓ | ✓ |  | ✓ | ✓ | ✓ |  | ✗ | ✓ | ✓ | ✓ |
|  | FUS-TB | ✗ | ✓ | ✓ | ✓ |  | ✓ | ✓ | ✓ |  |  |  |  |  |
|  | IB-TB | ✗ | ✓ | ✓ | ✓ |  | ✓ | ✓ | ✓ |  |  |  |  |  |
| Hamid et al., 2019 | COG-TB | ✓ | ✓ | ✓ | ✓ |  | ✓ | ✓ | ✓ |  | ✓ | ✓ | ✓ | ✓ |
|  | FUS-TB | ✓ | ✓ | ✓ | ✓ |  | ✓ | ✓ | ✓ |  |  |  |  |  |
| Wegelin et al., 2019 | COG-TB | ✓ | ✓ | ✓ | ✓ |  | ✓ | ✓ | ✓ |  | ✓ | ✓ | ✓ | ✓ |
|  | FUS-TB | ✓ | ✓ | ✓ | ✓ |  | ✓ | ✓ | ✓ |  |  |  |  |  |
|  | IB-TB | ✓ | ✓ | ✓ | ✓ |  | ✓ | ✓ | ✓ |  |  |  |  |  |
| Costa et al., 2019 | FUS-TB | ✗ | ✓ | ✓ | ✓ |  | ✓ | ✓ | ✓ |  | ✗ | ✓ | ✓ | ✓ |
|  | IB-TB | ✗ | ✓ | ✓ | ✓ |  | ✓ | ✓ | ✓ |  |  |  |  |  |
| Turkay et al., 2020 | COG-TB | ✗ | ✓ | ✓ | ✓ |  | ✓ | ✓ | ✓ |  | ✗ | ✓ | ✓ | ✓ |
|  | FUS-TB | ✗ | ✓ | ✓ | ✓ |  | ✓ | ✓ | ✓ |  |  |  |  |  |
| Zhang et al., 2020 | COG-TB | ✗ | ✓ | ✓ | ✓ |  | ✓ | ✓ | ✓ |  | ✗ | ✓ | ✓ | ✓ |
|  | IB-TB | ✗ | ✓ | ✓ | ✓ |  | ✓ | ✓ | ✓ |  |  |  |  |  |
| Yamada et al., 2020 | COG-TB | ✗ | ✓ | ✓ | ✓ |  | ✓ | ✓ | ✓ |  | ✗ | ✓ | ✓ | ✓ |
|  | FUS-TB | ✗ | ✓ | ✓ | ✓ |  | ✓ | ✓ | ✓ |  |  |  |  |  |
| Izadpanahi et al., 2021 | COG-TB | ✓ | ✓ | ✓ | ✓ |  | ✓ | ✓ | ✓ |  | ✓ | ✓ | ✓ | ✓ |
|  | FUS-TB | ✓ | ✓ | ✓ | ✓ |  | ✓ | ✓ | ✓ |  |  |  |  |  |
| Khoo et al., 2021 | COG-TB | ✗ | ✓ | ✓ | ✓ |  | ✓ | ✓ | ✓ |  | ✗ | ✓ | ✓ | ✓ |
|  | FUS-TB | ✗ | ✓ | ✓ | ✓ |  | ✓ | ✓ | ✓ |  |  |  |  |  |
| Guerra-Lacambra et al., 2023 | COG-TB | ✗ | ✓ | ✓ | ✓ |  | ✓ | ✓ | ✓ |  | ✗ | ✓ | ✓ | ✓ |
|  | FUS-TB | ✗ | ✓ | ✓ | ✓ |  | ✓ | ✓ | ✓ |  |  |  |  |  |
| Ito et al., 2023 | COG-TB | ✗ | ✓ | ✓ | ✓ |  | ✓ | ✓ | ✓ |  | ✗ | ✓ | ✓ | ✓ |
|  | FUS-TB | ✗ | ✓ | ✓ | ✓ |  | ✓ | ✓ | ✓ |  |  |  |  |  |
| Petov et al., 2023 | COG-TB | ✓ | ✓ | ✓ | ✓ |  | ✓ | ✓ | ✓ |  | ✓ | ✓ | ✓ | ✓ |
|  | FUS-TB | ✓ | ✓ | ✓ | ✓ |  | ✓ | ✓ | ✓ |  |  |  |  |  |

P = patient selection; I = index test; R = reference standard; FT = flow and timing.
✓ indicates low risk; ✗ indicates high risk; ? indicates unclear risk.

**sFigure 1.** Funnel plot to visually detect the presence of publication bias or heterogeneity for studies reporting cancer detection rates of clinically significant PCa

**sFigure 2.** Funnel plot to visually detect the presence of publication bias or heterogeneity for studies reporting cancer detection rates of insignificant PCa

**sFigure 3A.** Detection rate of clinically significant prostate cancer (csPCa)(3A) and indolent prostate cancer (ISUP GG 1) (3B) at targeted biopsy according to targeting technique. CsPCa at TB: clinically significant prostate cancer at targeted biopsy; N of patients: number of patients; ISUP 1 at TB: indolent prostate cancer at targeted biopsy.

A)

B)

**sFigure 4.** Detection rate of clinically significant prostate cancer (csPCa) at targeted biopsy according to targeting technique in randomized controlled studies. CsPCa at TB: clinically significant prostate cancer at targeted biopsy; N of patients

**sFigure 5.** Detection rate of ISUP Grade Group (ISUP GG) ≥2 at targeted biopsy according to target technique. CsPCa at TB: clinically significant prostate cancer at targeted biopsy; N of patients: number of patients; ISUP 1 at TB: indolent prostate cancer at targeted biopsy.

**sFigure 6.** Detection rate of clinically significant prostate cancer (csPCa)(6A) and indolent prostate cancer (ISUP GG 1) (6B) at targeted biopsy according to targeting technique in studies including all patients with PIRADS ≥3.

CsPCa at TB: clinically significant prostate cancer at targeted biopsy; N of patients: number of patients; ISUP 1 at TB: indolent prostate cancer at targeted biopsy.

A)

B)

**sFigure 7**. Detection rate of clinically significant prostate cancer (csPCa) at targeted biopsy according to targeting technique in biopsy naïve patients (7A) and patients with a previous negative biopsy (7B). CsPCa at TB: clinically significant prostate cancer at targeted biopsy; N of patients: number of patients.

6A)

6B)

**sFigure 8.** Detection rate of clinically significant prostate cancer (csPCa) at targeted biopsy according to targeting technique in patients undergoing transrectal targeted biopsy (8A) or transperineal targeted biopsy (8B). CsPCa at TB: clinically significant prostate cancer at targeted biopsy; N of patients: number of patients.

A)

B)

**sFigure 9.** Detection rate of clinically significant prostate cancer (csPCa) at targeted biopsy according to targeting technique in patients in patients with Peripheral zone (9A) or Transition Zone MRI lesions (9B). CsPCa at TB: clinically significant prostate cancer at targeted biopsy; N of patients: number of patients.

A)

B)

**sFigure 10.** Detection rate of clinically significant prostate cancer (csPCa) at targeted biopsy according to targeting technique in patients in patients with ≤10 mm (10A) or >10 mm (10B) MRI lesions. CsPCa at TB: clinically significant prostate cancer at targeted biopsy; N of patients: number of patients.

A)

B)

**sFigure 11.** Detection rate of clinically significant prostate cancer (csPCa) at a combination of targeted and Systematic Biopsy according to targeting technique. CsPCa at combined biopsy: clinically significant prostate cancer at combined standard+targeted biopsy (for patients undergoing in bore-TB only targeted cores are considered); N of patients: number of patients.
